# What is the role of metabolic syndrome and obesity for postoperative atrial fibrillation after coronary bypass grafting?

**DOI:** 10.1186/s12872-019-1130-3

**Published:** 2019-06-17

**Authors:** Ünsal Vural, Ahmet Arif Ağlar

**Affiliations:** Dr. Siyami Ersek Kalp ve Damar Cerrahisi Eğitim ve Araştırma Hastanesi Cardiovascular Surgery Clinic, Tıbbiye Cad. Haydarpasa Uskudar, 34668 Istanbul, Turkey

**Keywords:** Metabolic syndrome, Obesity, Atrial fibrillation, Coronary bypass grafting, Postoperative evaluations

## Abstract

**Background:**

Postoperative atrial fibrillation is the most common arrhythmia seen after cardiac surgery. We aimed to determine the effect of obesity and metabolic syndrome on postoperative atrial fibrillation, whether they are independent risk factors, and their effect level. We also analyzed the effect of atrial fibrillation on postoperative complications.

**Methods:**

In our clinic, 756 patients who underwent coronary artery bypass grafting between June 2010–September 2017 were evaluated retrospectively. Preoperatively, demographic characteristics, chronic diseases, body mass index, waist circumference measurements, and ejection fraction values of the cases were determined from file records. Perioperatively, cross-clamp and cardiopulmonary bypass times, intra-aortic balloon use, distal coronary bypass counts were determined. Postoperatively, complications, duration of intensive care unit and hospital stay, and mortality were evaluated. The patients were followed up with continuous 3-lead ECG monitorization on the postoperative first day and 12-lead ECG records once in a day on the remaining days. In the study, the first endpoint was the determination of atrial fibrillation and the second endpoint was the discharge time of the patient.

**Results:**

The rate of postoperative atrial fibrillation was 21.3%. Atrial fibrillation was seen in 33% of metabolic syndrome cases and in 38.5% of obese cases. Atrial fibrillation was seen in 23, 24 and 17% of cases using statin, ACE inhibitor and beta blocker, respectively. It was seen in 21% of smokers and 20% of the COPD cases. In the study, metabolic syndrome, diabetes mellitus, hypertension, and obesity, between the ages of 56–78 with Metabolic syndrome, were found to affect the development of postoperative atrial fibrillation (2.46), (2.3), (1.6), and (1.65) times, respectively. In cases with postoperative atrial fibrillation, infection and stroke were 1.45 and 8.85 times more frequent, respectively. Patients with metabolic syndrome were found to have 31% longer hospital stay, and 17% higher infection rate. In obese patients, hospitalization was 23.5% longer.

**Conclusions:**

Metabolic syndrome and obesity were found to be two independent risk factors for postoperative atrial fibrillation. If causes and mechanisms of postoperative atrial fibrillation are identified in planned cardiovascular interventions, we believe that cost of hospitalization and morbidity will be reduced.

## Background

The metabolic syndrome (MS) is known as a syndrome characterized by increased abdominal obesity, increased insulin resistance, decreased high-density lipoprotein (HDL), and elevated Low-density lipoprotein. In the United States, it is reported that its prevalence is more than 27% over 20 years of age and in women [1]. Kozan et al. reported that in Turkey, metabolic syndrome was seen in 33.9% of the population over 20 years of age and more frequently in women [2]. These data were obtained when the upper limit of the waist circumference is 102 cm for men and 88 cm for women. We believe that today’s accepted upper limits of 94 and 80 cm increase these rates. In MS, increased circulating cytokines secondary to metabolic disorders are thought to stimulate atrial fibrillation (AF). The incidence of AF is 0.4% in the general population, 30–40% after coronary artery bypass grafting (CABG), and 60% after valve surgery [3]. In the USA, it is known that AF-caused hospital admissions have increased by 66% over the last 20 years [4]. Postoperative atrial fibrillation (POAF) has been reported to increase hospital admissions in the US by ~ 30 days, resulting in an additional cost of $ 18,000–19,000 and a 2 or 3-fold increase in stroke risk [5]. Watanabe et al. demonstrated that metabolic syndrome is an independent risk factor for AF development even in the absence of diabetes and hypertension (HT), and is strongly associated with stroke, myocardial infarction (MI), and all-cause mortality [6].

In this study, we aimed to evaluate whether obesity and metabolic syndrome are independent risk factors among the risk factors affecting POAF in the light of literature. We also analyzed the effect of factors that stimulated POAF formation on postoperative complications and length of hospital stay.

## Methods

This study was performed at Dr. Siyami Ersek Thoracic and Cardiovascular Surgery Center Istanbul, Turkey. The study protocol was approved by the Health Sciences University Siyami Ersek Cardiovascular Surgery Hospital Ethics Committee decision dated 16/06/2015 and numbered (Number No: 28001928–501.07.01) and the patients were informed in writing. Informed consent was waived by Institutional Review Board owing to the study’s retrospective nature.

### Study population

**Fig. 1:**
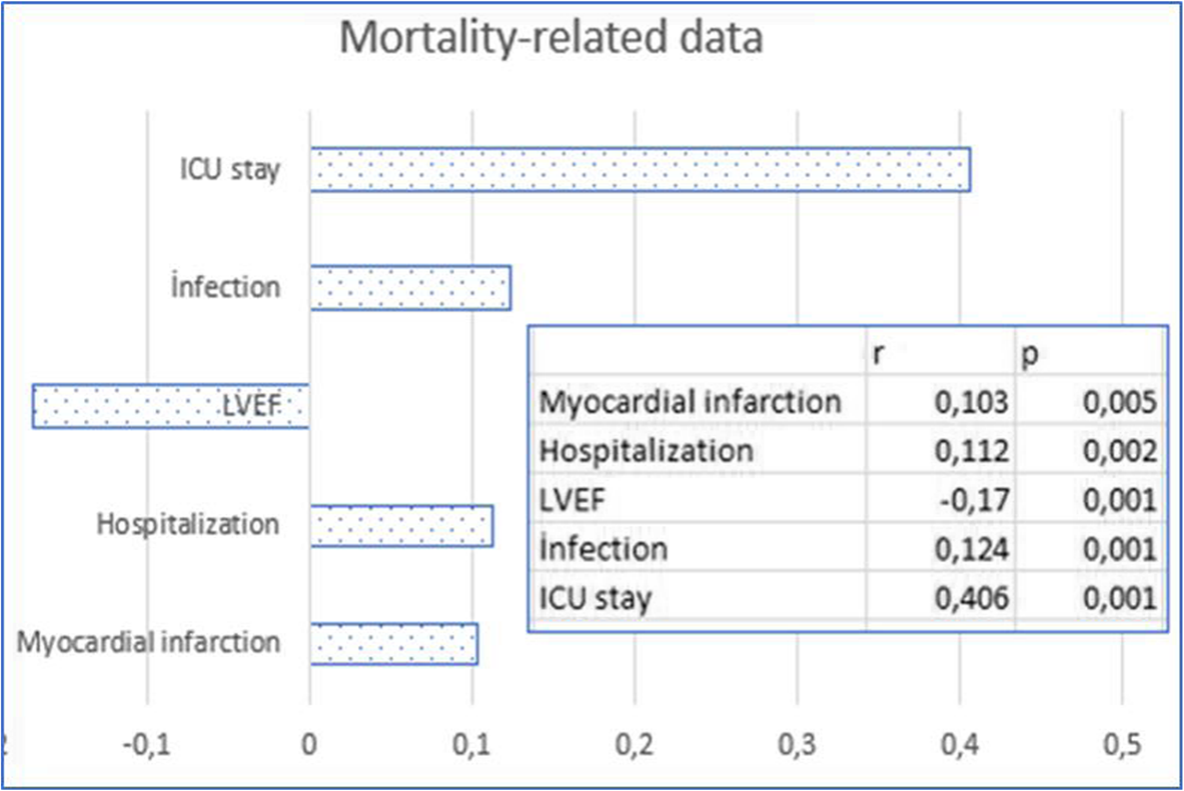
Significance status of the mortality associated factors and direction of the relationship

In our clinic, 756 patients who underwent isolated CABG between June 2010 and September 2017 were evaluated retrospectively. Patients with preoperative AF treatment (1), additional cardiac interventions (2), hyperthyroidism (3), moderate-to-severe liver (4) and renal (5) failure, malignancy (6) and those with no available record of body mass index (BMI) and waist circumference (WC) measurements were excluded. Table 1 shows the demographic characteristics of the cases [age, gender, drugs used (statin, beta blocker, Angiotensin converting enzyme inhibitors (ACE)), smoking, BMI, history of diabetes mellitus (DM), HT, chronic obstructive pulmonary disease (COPD) and post-myocardial infarction, measurements of triglyceride (TG), HDL, waist circumference and left ventricular ejection fraction (LVEF)]. Perioperatively, total and partial cardiopulmonary bypass (CPB) times, Intraaortic balloon pomp use, and the number of bypasses were determined. Postoperatively, infection, stroke, hemorrhage, duration of intensive care unit (ICU) and hospitalization and mortality were evaluated (Fig. 1). POAF formation of MS and obesity cases were compared to each other and to non-AF cases (Table 1).

**Table 1:** Statistical analysis of cases according to POAF and Metabolic Syndrome status (p^(a)^ = Independent sample t-test, p^(b)^ = Pearson chi-square test, *p* < 0.05 was considered significant.) CPB=Cardiopulmonary bypass, IABP = Intraaortic balloon pump, MI = Myocardial infarction, TG = Triglyceride, (+) = available, (−) = unavailable)

		Postoperative Atrial Fibrillation												
		Unavailable						Available						Total p^(b)^
		Metabolic Syndrome						Metabolic Syndrome						
		Unavailable		Available		Total	P^*(*b)^	Unavailable		Available		Total	P^(b)^	
		n	%	n	%			n	%	n	%			
Gender	Female	132	74,6	45	25,4	177	0,92	30	57,7	22	42,3	52	0,52	0,53
	Male	310	74,2	108	25,8	418		57	52,3	52	47,7	109		
Age	18–35	21	75,0	7	25,0	28	0,01	3	50,0	3	50,0	6	0,38	0,01
	36–55	185	76,4	57	23,6	242		21	45,7	25	54,3	46		
	56–78	236	72,6	89	27,4	325		63	57,8	46	42,2	109		
BMI	< 25	249	100	0	0,0	249	0,01	35	89,7	4	10,3	39	0,01	0,01
	25–30	173	83,6	34	16,4	207		42	70,0	18	30,0	60		
	> 30	20	14,4	119	85,6	139		10	16,1	52	83,9	62		
ACE Use	(−)	362	95,5	17	4,5	379	0,01	72	75,8	23	24,2	95	0,01	0,27
	(+)	80	37,0	136	63,0	216		15	22,7	51	77,3	66		
Statin Use	(−)	421	78,1	118	21,9	539	0,01	83	57,2	62	42,8	145	0,01	0,84
	(+)	21	37,5	35	62,5	56		4	25,0	12	75,0	16		
Beta-Blocker Use	(−)	302	75,3	99	24,7	401	0,41	63	52,1	58	47,9	121	0,38	0,05
	(+)	140	72,2	54	27,8	194		24	60,0	16	40,0	40		
Cigaret Use	(−)	296	73,6	106	26,4	402	0,60	61	55,0	50	45,0	111	0,73	0,74
	(+)	146	75,6	47	24,4	193		26	52,0	24	48,0	50		
Hypertension	(−)	406	95,8	18	4,2	424	0,01	83	84,7	15	15,3	98	0,01	0,01
	(+)	36	21,1	135	78,9	171		4	6,3	59	93,7	63		
Diabetes Mellitus	(−)	409	99,8	1	0,2	410	0,01	79	100,0	0	0,0	79	0,01	0,01
	(+)	33	17,8	152	82,2	185		8	9,8	74	90,2	82		
COPD	(−)	316	74,5	108	25,5	424	0,83	63	53,4	55	46,6	118	0,79	0,61
	(+)	126	73,7	45	26,3	171		24	55,8	19	44,2	43		
MI (passed)	(−)	390	74,7	132	25,3	522	0,52	73	52,9	65	47,1	138	0,48	0,49
	(+)	52	71,2	21	28,8	73		14	60,9	9	39,1	23		
IABP	(−)	415	73,7	148	26,3	563	0,18	81	54,0	69	46,0	150	0,97	0,48
	(+)	27	84,4	5	15,6	32		6	54,5	5	45,5	11		
		Ort.	ss	Ort.	ss	Ort.	p^(a)^	Ort.	ss	Ort	ss	Ort	P^(a)^	Total p^(a)^
TG (mg/dl)		170,0	33,2	200,4	56,3	185,2	0,01	169,2	34,3	187,6	48,4	178,4	0,01	0,96
HDL(mg/dl)		38,9	7,5	37,0	6,5	38,0	0,01	39,1	6,9	38,2	7,2	38,7	0,44	0,67
Total CPB (/min)		86,2	6,5	86,4	7,2	86,3	0,82	84,7	6,1	87,2	7,7	86,0	0,02	0,53
CCT(/min)		56,2	6,5	56,4	7,2	56,3	0,82	54,7	6,1	57,2	7,7	56,0	0,02	0,54
LVEF (%)		51,1	7,9	49,7	9,0	50,4	0,06	49,7	9,4	48,9	9,2	49,3	0,57	0,08
Hospitalization (/day)		7,8	1,6	8,3	1,9	8,1	0,01	8,7	1,5	9,1	2,1	8,9	0,11	0,01
CABG		2,9	0,4	2,9	0,4	2,9	0,85	2,8	0,4	2,9	0,4	2,8	0,18	0,39
Waist circumference(cm)		76,9	11,8	95,3	10,6	86,1	0,01	84,5	11,6	96,8	10,4	90,6	0,01	0,01

For the diagnosis of MS, diagnostic criteria proposed by MS Working Group of The Society of Endocrinology and Metabolism of Turkey (2005) were taken as basis [7]. Accordingly, to establish the MS diagnosis, in addition to presence of at least one of the such parameters as DM, impaired glucose tolerance or insulin resistance, having at least two of following parameters of (I) HT (systolic> 130 mmHg, diastolic> 85 mmHg or being under antihypertensive drug therapy), (II) dyslipidemia (TG > 150 mg/dl or HDL ≤ 40 mg/dl for male/50 mg/dl for female), (III) abdominal obesity (BMI > 30 kg/m^2^ or WC > 94 cm for male and > 80 cm for female) was required. The waist circumference was measured through the middle of the distance between the arcus costarium and the spina iliaca anterior superior. BMI was used for the detection of obesity.

### Follow up in hospital

The cases were followed by continuous Electrocardiography (ECG) monitoring for the first 24 h postoperatively while daily 12-lead ECG record was used for rhythm follow-up on other days. Patients with AF rhythm were monitored until the sinus rhythm was restored. In addition, ECG recording was taken in case of arrhythmia. Amiodarone hydrochloride loading and maintenance doses [8 ampoules (1200 mg) in 500 cc %5 Dextrose solution] were administered to patients who developed AF rhythm. Loading dose of 5–10 mg/kg was administered intravenously within the first 30 min. Maintenance dose was administered as 36–60 mg/h (900–1500 mg/day). Oral amiodarone (200 mg; 2 × 1) started after 24 h. Low-molecular-weight heparin (enoxaparin sodium 75 IU/kg) was given to patients with AF. Mechanical cardioversion was applied to the cases resistant to medical cardioversion within first 24 h. Postoperatively, all cases had continued to use the beta blocker, statin and ACE inhibitor used before the operation. In the study, the first endpoint was the detection of atrial fibrillation and the second endpoint was the discharge time.

### Statistical analysis

Continuous variables were expressed as a mean and standard deviation, while intermittent variables were expressed as number and percentage. Shapiro-Wilk test was used to determine whether the data were normally distributed. In the independent, continuous and normally distributed variables, Student’s t-test was used for binary comparisons whereas ANOVA F-test was used for triple or more comparisons. The Tukey posthoc test was used to analyze the difference between the three groups. Mann Whitney U-test was used for binary comparison of non-normally distributed variables while the Kruskal Wallis H-test was used for triple or more comparisons. Chi-square, Fisher exact and Yates continuity-correction tests were used for comparison and risk analysis of the nominal data of the two groups. The relationship between the groups was analyzed by the Pearson correlation test. Logistic regression was used to determine the effect of dependent and independent factors of POAF. The data were analyzed with the SPSS 17.0 statistical program for Windows. The number of samples was determined such that α ≤ 0.05 and β ≤ 0.20 in the study. Values of *p* ≤ 0.05 were considered significant.

## Result

Of the 756 cases (Mean age = 58.7 ± 12.8; male = 527) included in the study, 21.3% (*n* = 161) had POAF. In 88% of cases with POAF (*n* = 142), AF rhythm occurred within 1–5 days, postoperatively. In 98% (*n* = 158) of cases with POAF, sinus rhythm was restored within 1–3 days.

**Fig. 2:**
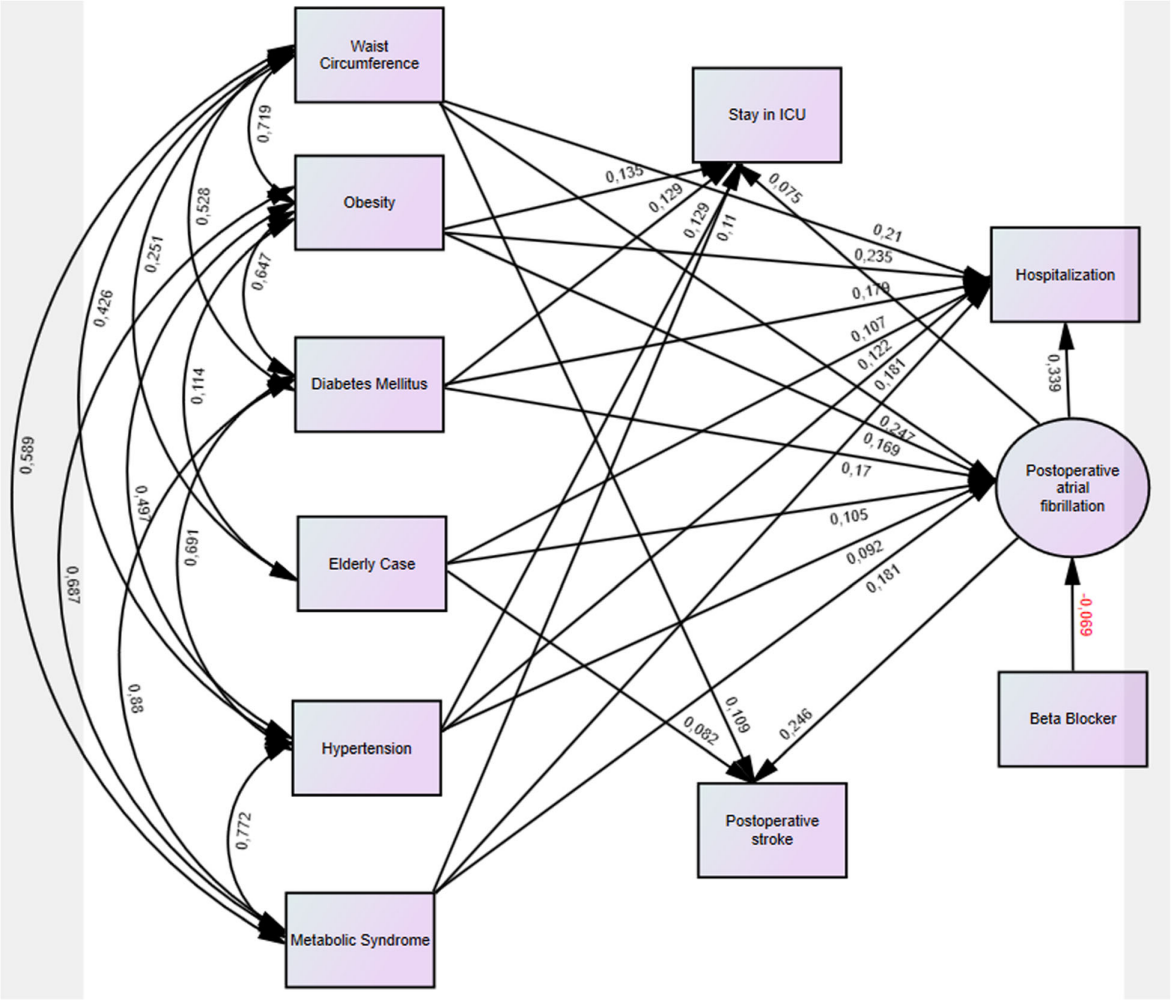
The demographic characteristics of the patients were positively correlated with each other and with postoperative atrial fibrillation. The picture shows the direction of the interaction and the correlation coefficients r. Similarly, demographic features which were shown in the picture, and atrial fibrillation, were found to be related positively with both intensive care and hospital stay

**Fig. 3:**
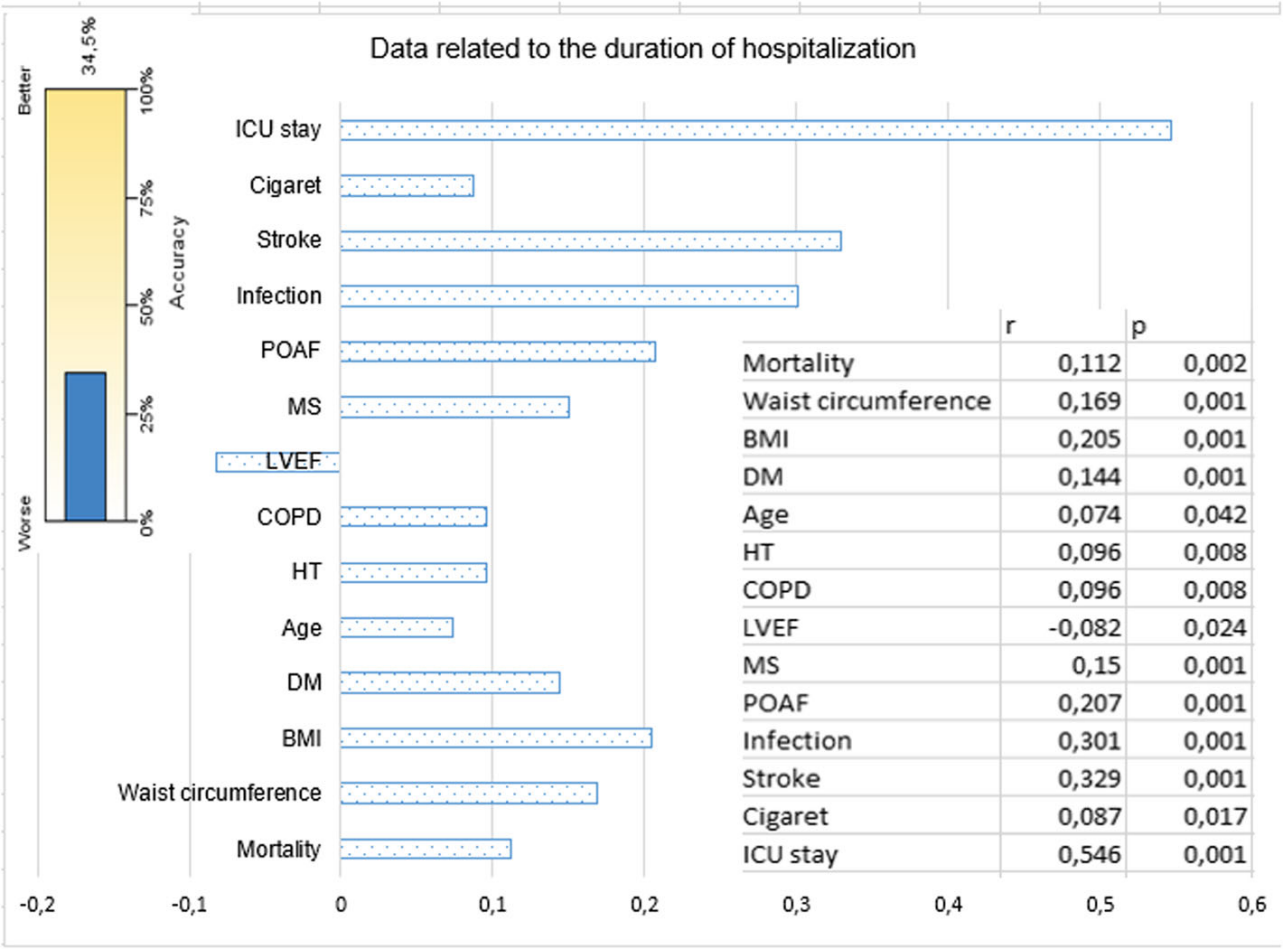
The parameters that affect the length of hospitalization, *p*-values, r-correlation coefficients showing the direction and rate of effect, and significance levels are shown. Data reveals 34.5% of all factors affecting the length of hospital stay

AF rhythm developed in 23% (*n* = 52) of female cases and in 21% (*n* = 109) of males. Distribution by sex was not significant (*p* = 0.532). The difference was significant in obese cases (BMI ≥ 30 kg/m^2^; *p* = 0.001; Table 1). It was found that 33% (*n* = 74; *p* = 0.001) of cases with MS, 23% (*n* = 16; *p* = 0.840) of statin users, 24% (*n* = 66; *p* = 0.275) of ACE inhibitor users, 17% (*n* = 40; *P* = 0.059) of beta-blocker users, 21% (*n* = 50; *P* = 0.739) of smokers and 20% (*n* = 43) of cases with COPD developed POAF (Table 1). The development of POAF was found to increase the length of hospitalization by 20.7% (*p* = 0.001). POAF development was associated with BMI by 16.9% (*P* = 0.001), with WC by 24.7% (*P* = 0.001), and with age by 7.3% (*p* = 0.044; Figs. 2 and 3; Table 1). The mean of waist circumference was 90.1 ± 12.6 cm in the cases with POAF while was 81.6 ± 14.0 cm in the other cases. The difference was significant (*p* = 0.004). Incidence of postoperative atrial fibrillation was increased by 2.46 times (*n* = 74; OR = 2.46 95% CI = 1.7–3.5) in cases with MS, by 2.3 times (*n* = 82; OR = 2.3; 95% CI = 1.61–3.3) in cases with DM, and by 1.6 times (*n* = 63 OR = 1.59; 95% CI = 1.1–2.3) in hypertensive cases. The postoperative infection and stroke were found to be 1.45-fold (*n* = 16; OR = 1.45 95% CI = 0.794–2.66; Table 2), and 8.8-fold (*n* = 23; OR = 8.84% 95 CI = 4.2–18.5) more frequent in MS cases, respectively. There was no relationship between mortality and POAF.

**Table 2:** The analysis of effect of preoperative demographic characteristics of patients on mortality, hospitalization, ICU stay, stroke, infection and bleeding. (ACE = angiotensin converting enzyme, HT = hypertension, COPD = Chronic Obstructive Lung Disease) (p^b^ = Pearson Chi-square test, p^a^ = Independent Sample t-test, p^c^ = One Way Anova test)

		Mortality		p^b^	Hospitalization		p^a,c^	ICU Stay		p^a,c^	Strok		p^b^	Infection		p^b^	Bleeding		p^b^
		Not	Yes		Ort.	Ss		Ort.	Ss		Not	Yes		Not	Yes		Not	Yes	
Gender	Female	225	4	0,54	8,2	1,94	0,41	1,9	1,8	0,66	217	12	0,52	209	20	0,47	223	6	0,42
	Male	514	13		8,1	1,71		1,9	1,7		505	22		489	38		507	20	
ACE	(−)	465	9	0,40	8,1	1,77	0,04	1,8	1,7	0,27	452	22	0,80	444	30	0,07	457	17	0,77
	(+)	274	8		8,3	1,79		2,0	1,8		270	12		254	28		273	9	
Statin Use	(−)	667	17	0,35	8,1	1,76	0,15	1,9	1,8	0,44	651	33	0,18	633	51	0,49	661	23	0,73
	(+)	72	0		8,4	1,94		1,8	0,8		71	1		65	7		69	3	
Beta Blocker	(−)	510	12	0,89	8,1	1,72	0,80	1,9	1,7	0,74	496	26	0,34	482	40	0,99	505	17	0,68
	(+)	229	5		8,2	1,91		1,9	1,7		226	8		216	18		225	9	
POAF	(−)	581	14	0,71	8,0	1,72	0,01	1,9	1,7	0,24	584	11	0,01	553	42	0,24	573	22	0,45
	(+)	158	3		8,9	1,82		2,0	1,7		138	23		145	16		157	4	
Metabolic Syndrome	(−)	519	10	0,31	8,0	1,64	0,01	1,8	1,5	0,02	510	19	0,07	496	33	0,02	509	20	0,43
	(+)	220	7		8,6	2,02		2,1	2,1		212	15		202	25		221	6	
HT	(−)	513	9	0,15	8,0	1,70	0,01	1,8	1,6	0,01	501	21	0,35	486	36	0,23	502	20	0,38
	(+)	226	8		8,4	1,94		2,1	2,0		221	13		212	22		228	6	
Diabetes Mellitus	(−)	479	10	0,61	8,0	1,63	0,01	1,8	1,5	0,01	472	17	0,07	458	31	0,06	473	16	0,73
	(+)	260	7		8,5	1,98		2,1	2,0		250	17		240	27		257	10	
BMI	< 25	283	5	0,39	7,8	1,46	0,01^c^	1,8	1,6	0,06^c^	278	10	0,27	282	6	0,01	281	7	0,49
	25–30	262	5		8,2	1,78		1,8	1,6		256	11		242	25		256	11	
	> 30	194	7		8,7	2,06		2,1	2,0		188	13		174	27		193	8	
Cigaret Use	(−)	502	11	0,78	8,3	1,95	0,01	2,0	1,9	0,01	487	26	0,27	470	43	0,29	492	21	0,15
	(+)	237	6		7,9	1,35		1,7	1,3		235	8		228	15		238	5	
COPD	(−)	527	15	0,13	8,3	1,93	0,01	2,0	1,9	0,01	514	28	0,16	496	46	0,18	522	20	0,55
	(+)	212	2		7,9	1,29		1,6	1,1		208	6		202	12		208	6	
Age	18–35	32	2	0,20	8,0	2,24	0,12^c^	2,1	1,8	0,52^c^	33	1	0,07	32	2	0,03	31	3	0,02
	35–55	280	8		8,0	1,67		1,8	1,6		281	7		275	13		277	11	
	56–78	427	7		8,3	1,81		1,9	1,8		408	26		391	43		422	12	
Waist circumference (mean/cm)		Mean		p^a^							Mean		p^a^	Mean		p^a^	Mean		p^a^
		83	88	0,20							83	91	0,01	83	90	0,01	83	86	0,40

**Fig. 4:**
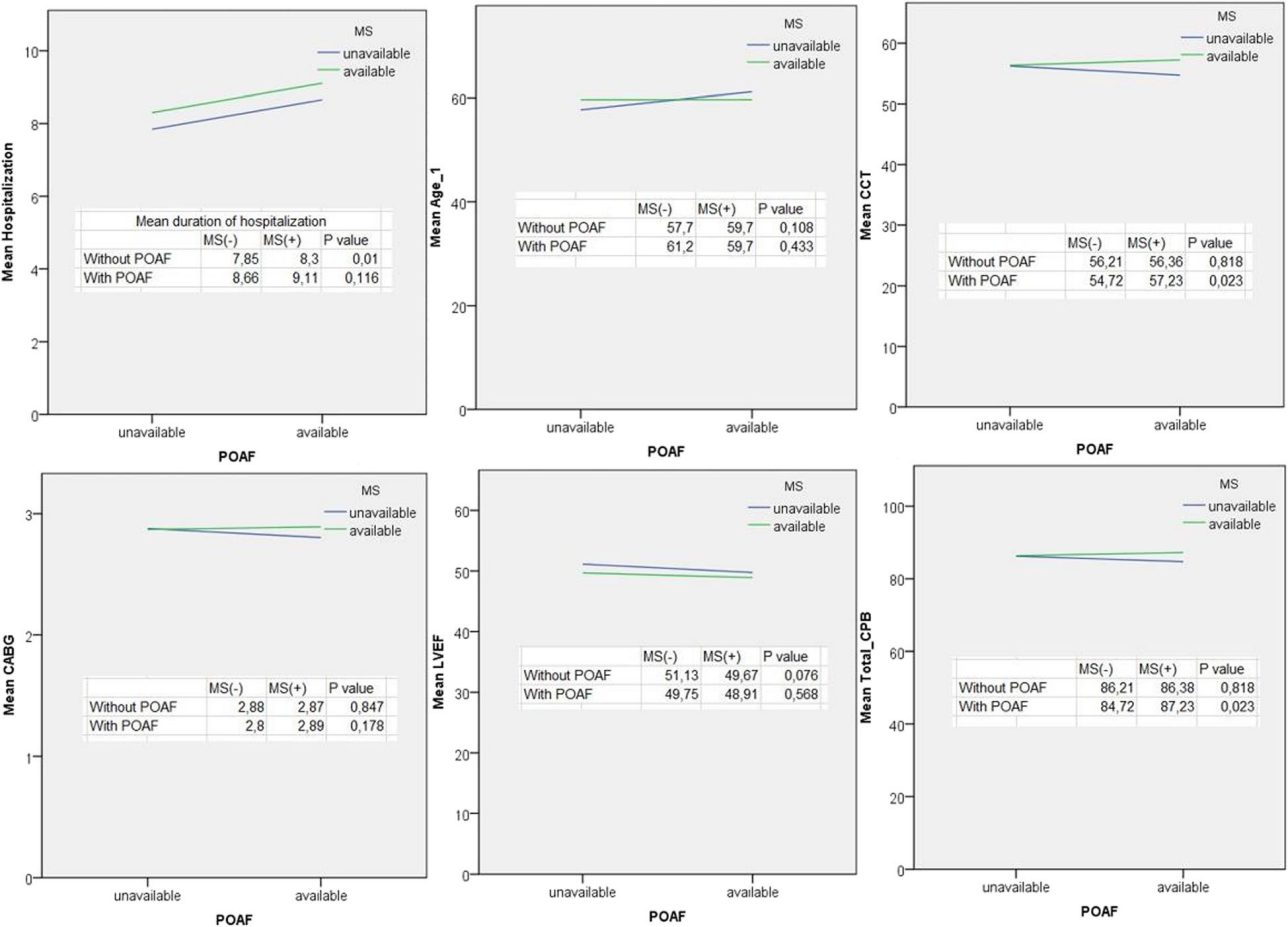
Hospitalization duration, age, Cross clamp time (CCT, total CPB duration, mean CABG number and LVEF were compared in cases with and without POAF according to MS status. Total CPB and CCT times were significantly longer in POAF and MS cases. In addition, MS alone extended the length of hospitalization significantly

**Fig. 5:**
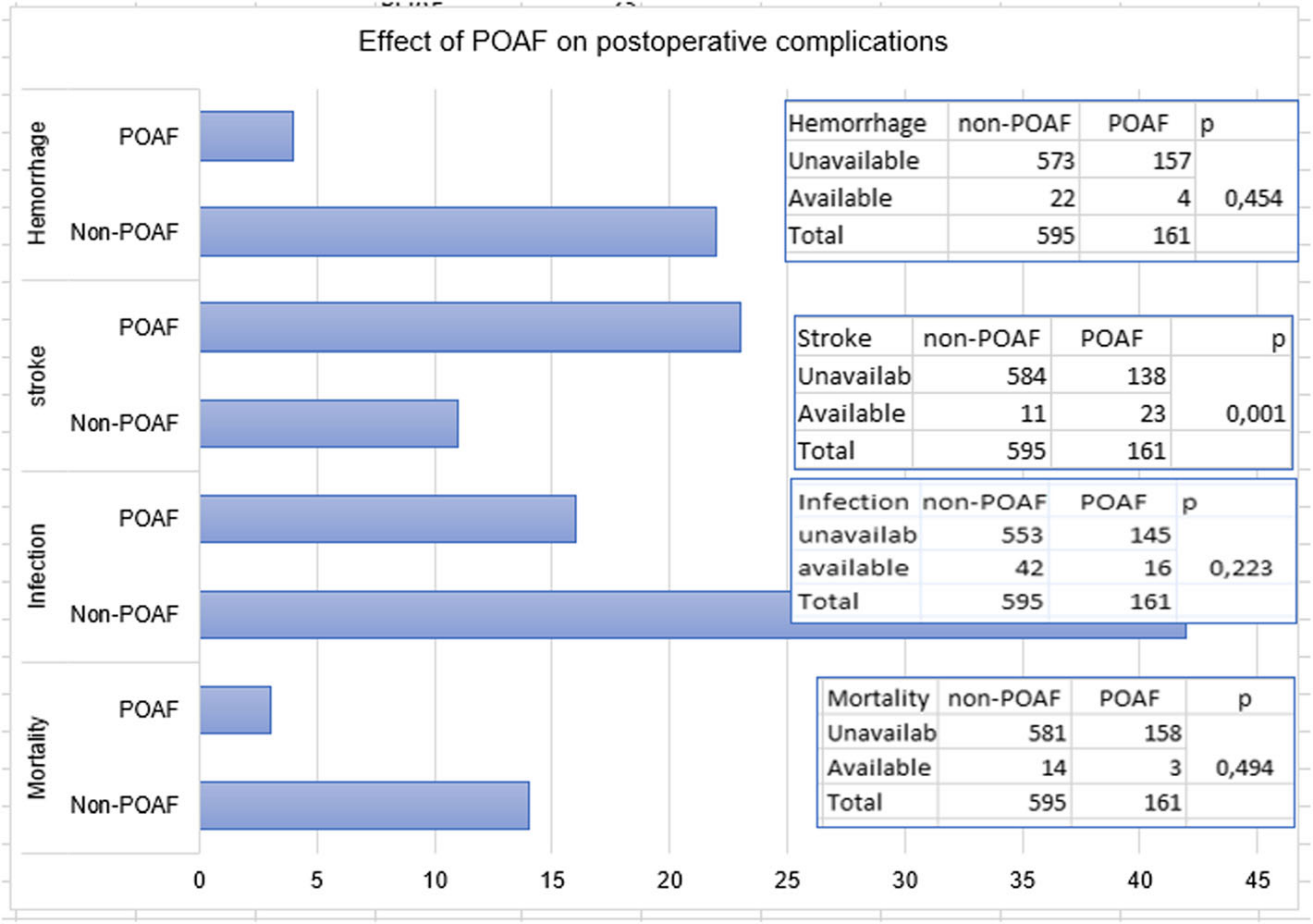
Graphical analysis of the effect of POAF on postoperative complications. POAF affected only the stroke rate significantly

**Fig. 6:**
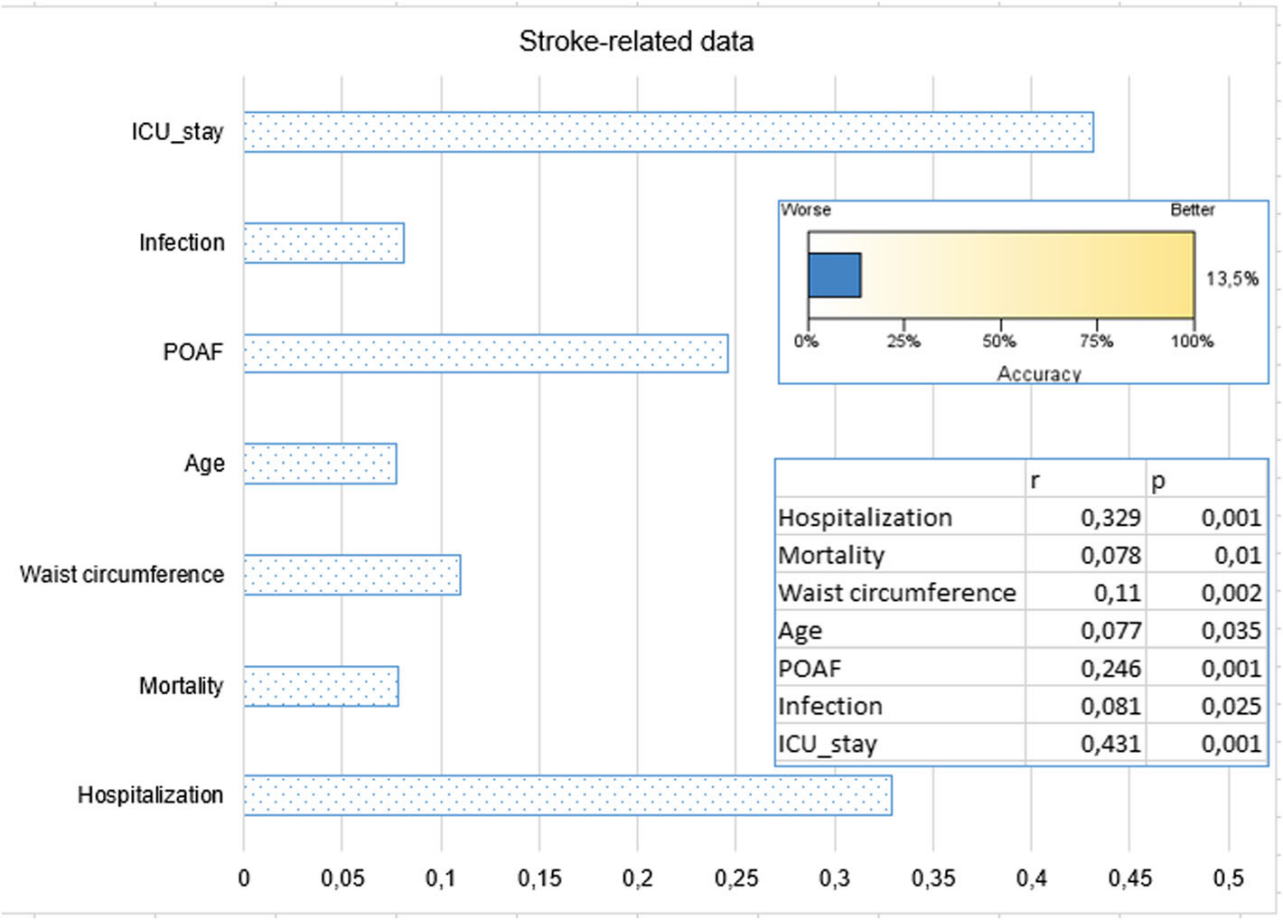
When all factors are considered, a graphical representation of the ratios of stroke-related data (*r* = correlation coefficient). It reveals only 13.5% of the factors associated with stroke status

MS was present in 29.5% of the female cases (*n* = 67; *p* = 0.7661). Since MS was frequent in advanced age patients, 31% (*n* = 135) of the POAF cases were in the 56–78 age group. POAF occurred in 46% of cases with MS (*n* = 74; *p* = 0.001; Table 2). When cases with MS who developed POAF were evaluated alone, lengths of hospitalization and intensive care unit stay were found to be increased by 18.1% (0.6/day) and by 11% (0.3/day), respectively (Figs. 4, 5 and 6). 43% of postoperative infections (*n* = 25; *p* = 0.024; OR = 1.86 95% CI = 1.08–3.2) and 44% of strokes (*n* = 15; OR = 1.9 95% CI = 0.95–3.8) were found to be occurred in cases with MS (Table 2; Fig. 5). Mortality was found to be 1.7 times higher in cases with MS (*n* = 7; *p* = 0.310; OR = 1.65 95% CI = 0.62–4.39; Table 2). However, it was not statistically significant.

Of the 201 patients (26.6%) who were obese according to BMI, 30.8% was found to have POAF (30.8%; *n* = 62; *p* = 0.001) and the difference was significant compared to normal cases. 13.4% of postoperative infections (*n* = 27; *p* = 0.001) were seen in this group. There was a significant positive correlation between postoperative infection and BMI (17%; *p* = 0.001). However, there was a low positive correlation with stroke (*p* = 0.292; Figs. 2 and 6). Mortality was higher than the other groups (3.5%) but the relationship was not significant (*p* = 0.386; Fig. 1). In the obese cases, the hospitalization period was found to be 23.5% longer. Obesity increased the risk of AF by 1.65-fold (*n* = 62; OR = 1.65 95% CI = 1.4–1.9) in the 56–78 age group.

There was a positive correlation between age and AF (*p* = 0.010; Fig. 2). In the cases without MS, the rate of AF, which was 25% in the 18–35 age group increased to 27.4% in the 56–78 age group. However, in cases where metabolic syndrome was added, these rates increased up to 50% (Table 1). However, the number of cases with both metabolic syndrome and atrial fibrillation was low in the 18–35 age group due to insufficient number of cases with coronary bypass. POAF rates in cases with MS were between 42 and 54% in the 36–56 and 56–78 age groups where the number of cases were enough. When all cases considered, AF developed in 17.6% of the cases (*n* = 6) belonging to the 18–35 age group, in 16% of the cases (*n* = 46) belonging to the 36–55 age group, and in 25% of the cases (*n* = 109) belonging to the 56–80 age group. 67.7% of cases with AF were in the 56–78 age group (*p* = 0.012; Table 1). The rate of postoperative infection in the same age group was 9.9% (*p* = 0.026). Effect of the age on the length of hospitalization in the POAF developed cases was found to be 10.7% (*p* = 0.012) and that was statistically significant. There was 6% relationship between stroke and age but not significant (*n* = 26; *p* = 0.058). In addition to higher MS incidence in elderly patients, there was also a positive relationship between POAF and age in the cases without MS (*p* = 0.02; *r* = 0.10).

When the patients with the metabolic syndrome alone were evaluated in terms of AF occurrence, the increase in AF risk was not significant in the 18–35 age group (*p* = 0.223), was 3.7 times in the 36–55 age group (*p* = 0.001; OR = 3.86 95% CI = 2.01–7.41), and was 1.9 times in the 56–79 age group (*p* = 0.001; OR = 1.96 95% CI = 1.23–3.04; Table 1). Although these results were a discordance with age increase, this was a result of the shadowing of the age by metabolic syndrome. Hypertension was diagnosed in 31% (*n* = 234) of the cases. In 26.9% (*n* = 63; *p* = 0.011; Table 1) of hypertensive cases, POAF developed and the relation was significant. MS was detected in 82.9% of hypertensive patients (*n* = 194). The lengths of hospitalization and intensive care unit stay of these cases were longer than other cases by 9.6 and 8.9%, respectively. At the same time, BMI values were significantly higher in these cases (*p* = 0.001). Hypertension was found to increase the AF incidence by 1.6-fold (*p* = 0.011; OR = 1.59%95 CI = 1.11–2.29), infection incidence by 1.4-fold (*p* = 0.232; OR = 1.43%95 CI = 0.80–2.43), and mortality by 2-fold (%3.4; *p* = 0.146; OR = 2.01%95 CI = 0.77–5.29).

When the factors affecting the duration of hospitalization were examined (Table 2), the POAF was found to be the major contributing factor by 34%. The effect of such factors as BMI and WC on the length of hospital stay was 21–23% (Fig. 2). Cases with DM and MS were found to have longer duration of hospitalization by 18% (Figs. 2 and 3). Duration of hospitalization was significantly prolonged by infection (23.2%; *p* = 0.001), stroke (24.5%; *p* = 0.001), low LVEF (%7; *p* = 0.035), and age (%11.3; *p* = 0.002; Figs. 2 and 3).

## Discussion

Many studies, report 15–40% AF incidence within 1–5 days after surgery [8]. Its clinical significance depends on the underlying factor. Within the first 2 h, 30% of POAF cases recover spontaneously. It has been reported that 25–80% of POAF cases recover in 24 h by using only the digoxin [9]. Mathew et al. reported that POAF was affected by older age, male gender, HT, AF history, heart failure, valvular disease, COPD, preoperative digoxin use, and non-use of beta-blockers preoperatively [10]. The incidence of POAF has been reported be more in cases whose surgery encompasses pulmonary vent placement and/or bicaval cannulation [10]. Since our series consisted of coronary artery bypass cases, bicaval cannulation and pulmonary venting were not performed. 88% of the POAF cases developed within postoperative 1-5th days and 98% of these recovered within 1–3 days. As the LVEF value decreased, the incidence of POAF increased but the relationship was not significant (Figs. 3 and 4). Significant results may be obtained, however, in the series in which very low LVEF cases are included. In our cases, it was found that DM, MS, obesity, and advanced age had affected POAF positively. On the other hand, significant negative correlation (6.9%) between POAF and beta-blocker use was detected (Table 1; Fig. 2). However, statin and ACE inhibitor use were not found to be correlated with POAF (*p* > 0.05). Mathew et al. [10] have found an association between ACE inhibitor or statin use and POAF, which can be explained by the fact that the number of cases using these drugs was higher than our count. It was found that POAF significantly increased the length of hospitalization and ICU stay, and the stroke rate (Figs. 3, 4 and 6).

Roffman et al. reported that as the number of bypass grafts or the CPB duration increases, the rate of arrhythmia increases [11]. As Bannister et al. reported, the increase in CPB duration deranges the mechanism by which glucose is transported into the cell and thus blood glucose level is elevated. As a result, metabolic acidosis occurs. When the patient is warming up, the insulin response increases but hyperglycemia persists for another 1–2 h. In addition, the metabolism of thyroid hormones is affected and the level of triiodothyronine (T_3_) falls [12]. In our study, there was no difference between the number of bypasses, and total and partial CPB durations of the cases (Table 1, Fig. 4). If studies with groups with different total and partial CPB durations are performed, it could be analyzed whether this difference is a factor affecting POAF or not.

Studies have reported 34% prevalence of abdominal obesity in the population of 20 years of age and over in Turkey [13]. Although abdominal obesity is an important indicator of insulin resistance, obesity may not be present in some of the metabolic syndrome cases with insulin resistance. As the BMI increases, there is a gradual increase in left atrial dimensions. Ducceschi et al. reported that they found higher frequency of AF and left atrial dilatation in a series of 150 cases with BMI ≥30 kg/m^2^ [14]. In atrial biopsies of patients with atrial fibrillation, inflammatory mediators were detected high. This may explain the development of AF from postoperative inflammation [15]. It is believed that abdominal obesity increases the level of inflammatory cells and facilitates the development of AF with the released mediators. Adipose tissue is an active endocrine organ that secretes many hormones like leptin, resistin, adiponectin, and cytokines (TNF-alpha, IL-6, IL-8). Released cytokines cause systemic inflammation and affect insulin resistance and pulmonary functions [16]. In our study, we found that both high BMI measurements and high WC scores affected POAF significantly (*p* = 0.001). The mean WC value was 90.1 ± 12.6 cm in the POAF developed cases while it was 81.6 ± 14 cm in the cases that had not developed POAF (Table 2). Although WC values were higher in cases with postoperative complications such as mortality, infection, bleeding and stroke, it was found to be significant only in cases developing stroke and infection (Table 2).

The body mass index is an indicator of total fat accumulation and does not represent the distribution of fat or metabolic distress. It has also been reported in previous studies that obesity is defined by BMI and is an independent risk factor for POAF [17]. There have been recent reports that conclude obesity has no effect on POAF [17]. Differences between studies may be related to the heterogeneous distribution of fat and the rate of accumulation of cardiotoxic metabolites. In parallel with most centers, our opinion is that WC measurement is more reliable than BMI as a diagnostic parameter for MS. In our study, we found that the incidence of POAF was affected by obesity by 1.65-fold while by MS by 2.46-fold (Fig. 2).

In MS, endothelial dysfunction has been reported to develop before clinical symptoms occur [18]. This may contribute to the view that endothelial dysfunction plays a role in the development of POAF in MS. Almassi et al. reported 2-fold higher hospital mortality (3% versus 6%) in post-operative AF cases [19]. The 6-month mortality rate was reported to be 4.7% vs 9%. We think that this mortality difference is due to the factors that paved the way for POAF development in addition to POAF. Since the duration of follow up in our study was limited to the length of hospitalization, it was not possible to determine the mid and long-term mortality. However, hospital mortality was not significantly different between POAF and Non-POAF cases (2.2%). Mortality of cases diagnosed with MS was found to be increased by 2.4 times (Figs. 1 and 5) with POAF, whereas by 1.7 times without POAF. As it is understood from these results, it would be incomplete to think that only POAF increases mortality. POAF also increases postoperative morbidity. In cases with MS, POAF was found to increase the length of the hospitalization by 31% (*p* = 0.001). In our study, we found that MS increased the infection and stroke incidence by 1.9-fold while the stroke was more frequent in patients in MS cases who developed POAF (Figs. 2, 3 and 6).

Geographical region and race were found to be effective in the development of POAF as following regional incidences indicate: Middle East (41.6%), USA (33.7%), Europe (34%), Canada (36.6%), South America (17.4%), Asia (15.7%) [20]. This differentiation may be related to the incidence of MS as well as indicating that the white race is more prone to POAF. However, there is a need for meta-analyzes of large series and cohort studies in which the accompanying factors are examined. In our cases, the incidence of POAF is 21.3%, similar to the geographical distribution, though the 32.6% incidence rate of POAF in MS cases s differentiated from the literature data. In addition, the prevalence rate of MS in our series (30%) was similar to the prevalence of MS in the literature (33%) [2].

The incidence of AF in the general population (0.4–1%) has been reported to increase by age such that it is 8% over the age of 80 [21]. In elderly patients, the increase in the rate of POAF is also due to changes in the cardiac fibrosis and atrial dilation [22]. In our series, in the cases without MS, the rate of AF which was 25% in the 18–35 age group increased to 27.4% in the 56–78 age group. However, in cases where metabolic syndrome was added, these rates increased up to 50% (Table 1). The rate of postoperative infection in the same age group was 9.9% (*p* = 0.026). Effect of the age on the length of hospitalization in the POAF developed cases was found to be 10.7% (*p* = 0.012) and that was statistically significant (Table 1). We think that besides the degree of atrial fibrosis increasing with age, MS which is more common in older ages is also effective on the increase of POAF incidence. In addition to such preventable causes as surgical dissection, manipulation, pericardial injury, pericarditis, left ventricular dysfunction and atrial dilation due to intraoperative volume overload, electrolyte irregularities, and blood transfusion, techniques for administering the cardioplegia and inadequate atrial cooling could activate the complement system through oxidative stress-induced release of inflammatory mediators [23]. Supporters of this theory argue that the use of anti-inflammatory drugs together with corticosteroids and statins reduces the rate of POAF [10, 24]. Since all our cases had similar temperatures and durations of CPB, we believe that the effect of such confounding factors on the results was not significant.

It has been reported that 60% of postoperative AF cases have HT [24]. Patti et al., report that HT is an independent risk factor for POAF [25]. In our study, the AF rate (27%) in the cases with HT was found to be significant. Hospital mortality in those with HT was twice as high. Blood pressure control can be an important strategy in preventing AF.

We observed that the incidence of stroke was 8,85 times as high (Figs. 2, 3 and 6) in patients with atrial fibrillation. Our treatment strategy was to restore hemodynamic stability, prevent thromboembolism, and eliminate metabolic problems. The choice of anticoagulant treatment for sustaining AF cases was done according to the CHA2DS2VASC scoring system. Guidelines issued by the ESC in 2010 recommended the use of the CHA2DS2VASC scoring system [26]. By utilizing this scoring system created with large series, the expected risk of thromboembolism is calculated, and the appropriate anticoagulant treatment is determined.

## Limitations of the study

In our study, in cases with obesity and MS, left atrial diameter, a determinant of POAF, was not measured. Left atrial dilation, though an independent risk factor, may be responsible for some of the effects of MS or obesity on POAF. This can be determined by detailed echocardiographic examination. However, we believe that the interaction is not much since our cases did not have valve problems and the COPD patient count was low.

In the analysis of the effect of clinical factors on the POAF in the 18–35 age group, the absence of association may be due to the low number of samples in the group. In this group, the low number of cases was since atherosclerosis was rarely seen in this age group and PTCA method (stenting) was preferred in the treatment. However, this number, which we could still reach, was insufficient when divided into groups, but as a single group, it was enough for analysis. Patients with MS and obesity were hospitalized longer. This may lead to a prejudice that the capture rate of the POAF could have been affected.

## Conclusion

In our study, it was observed that POAF was common in obesity, MS, HT, DM and elderly patients and that it significantly prolonged hospital stay. Although AF incidence was lower in patients using beta blocker, it was not significant. In POAF cases, stroke rate was 8,85 times higher. POAF was found to be positively correlated with WC and BMI measurements. Obesity was found to be one of the important risk factors for POAF independent of MS.

Metabolic syndrome and obesity are important factors that decrease the quality of life by increasing the incidence of POAF after coronary bypass surgery. If the causes and mechanisms of POAF are determined, we believe that by reducing the factors leading to atrial fibrillation, hospital cost and morbidity will be reduced, in planned cardiovascular interventions.

## Data Availability

The datasets used and analyzed during the current study are available from the corresponding author on reasonable request.

## Abbreviations

ACE: Angiotensin converting enzyme inhibitor
AF: Atrial fibrillation
BMI: Body mass index
CABG: Coronary artery bypass grafting
CCT: Cross clamp time
COPD: Chronic obstructive pulmonary disease
CPB: Cardiopulmonary bypass
DM: Diabetes mellitus
ECG: Electrocardiography
HDL: High-density lipoprotein
HT: Hypertension
IABP: Intraaortic balloon pump
ICU: Intensive care unit
LVEF: Left ventricular ejection fraction
MI: Myocardial infarction
MS: Metabolic syndrome
POAF: Postoperative atrial fibrillation
TG: Triglyceride
WC: Waist circumference
